# Medical risk factors of diabetes mellitus among professional drivers

**DOI:** 10.1186/2251-6581-12-23

**Published:** 2013-06-01

**Authors:** Nazanin Izadi, Maryam Malek, Omid Aminian, Maryam Saraei

**Affiliations:** 1 Center for Research on Occupational Diseases, Tehran University of Medical Sciences, Tehran, Iran; 2 Occupational Health Office, Tehran University of Medical Sciences, Tehran, Iran; 3 Occupational Sleep Research Center, Tehran University of Medical Sciences, Tehran, Iran

**Keywords:** Professional drivers, Diabetes mellitus

## Abstract

**Background:**

Road transport drivers are one of the professional groups whose activities have a strong impact of public safety. In view of the natural professional activity, the drivers are at a higher risk of obesity, hypertension and hyperlipidemia, and carbohydrate metabolism disorders such as diabetes mellitus.

**Materials and methods:**

Medical documentation was the source of data for the reported study. It derived from medical examinations of 1903 drivers applying for driving license.

**Results:**

Hyperglycemia was found in 52.1% of the drivers, 9.1% of them were in diabetic stage, and with HbA_1_C criteria 77.6% of these drivers were in this stage. Excessive body weight was recorded in 65.6% of the study population, 44.8% were diagnosed with overweight and 20.8% with obesity. High blood pressure was recorded in 16.4% of drivers.

**Conclusion:**

High prevalence of excessive body weight and high blood pressure and hyperlipidemia are risk factors for diabetes mellitus in professional drivers that indicates a need to undertake multidimensional actions target on this particular profession and involving various health care sectors. Prophylactic and detailed pre-placement examinations should be considered, depending on the rate and the intensity of disorders. These should be coupled with an introduction of primary and secondary prophylactic activities and monitoring of relevant treatment.

## Introduction

[1] Diabetes mellitus (DM) refers to a group of common metabolic disorders in which a person has high blood sugar, either because the pancreas does not produce enough insulin, or because cells do not respond to the insulin that is produced. Several distinct types of DM are caused by a complex interaction of genetics and environmental factors. The prevalence of this disease has dramatically increased in the recent two decades. It is reported that this prevalence has increased from 30 million in 1985 to 285 million in 2010 [1].It is forecast that by 2030, 77.6% of diabetic patients will be from the developing countries [2,3]. Diabetes has increasingly become a great concern in the developing countries and Iran is no exception.

90-95% of diabetics those who are diagnosed after the age of 30 are affected by type 2 diabetes. It is reported that the prevalence of type 2 diabetes is increasing more rapidly, a trend which is claimed to be caused by the increased rate of obesity, sedentary lifestyle, and aging population. Fortunately however, this rampant disease can be prevented and managed through lifestyle modifications and medication therapy [1]. The prevalence of type 2 diabetes is reported to be 1.2%-14.6% in Asia, 4.6 to 40% in the Middle East, and 1.3 to 14.5% in Iran; and the highest prevalence is reported in some islands of the Pacific Ocean and the Middle East [2-6].

Drivers are among those professionals whose job and health status greatly influences the public safety. Any health issue that affects drivers may result in an increased risk for road accidents. Moreover, such problems can influence their performance, and cause sickness absenteeism, thereby posing great financial burden to the society [7]. Both complications of diabetes and medications’ side effects can affect driving skills. Diabetic neuropathy and retinopathy are two common complications which can cause muscle weakness or even amputation. Moreover, treatment of diabetes can result in hypoglycemia, which in turn, may lead to increased reaction time, imbalance and loss of consciousness [8].

In Iran, all drivers need to undergo periodic medical examinations defined by occupational health regulations. These periodic exams are administered by the Ministry of Health and Medical Education of the Islamic Republic of Iran and drivers need to carry a health card indicating that they have passed these tests all the time when driving [9].

In one study carried out on Hong Kong professional drivers, the prevalence of diabetes was 8.1% [10]; while in another study, this prevalence was 7% [11].

It can be suggested that drivers are faced with some health hazards in their job, such as stress, sitting for long periods, night and rotatory shifts put them at a higher risk for obesity and hypertension which are well-known risk factors for diabetes [12].

In Iran, 500,000 professional drivers are employed on a full time basis, a cohort which constitutes a great proportion of Iranian population [13]. In our country, to our knowledge, there have not been sufficient studies carried out on the prevalence of diabetes and its risk factors such as hypertension, hypertriglyceridemia, and obesity in drivers with a representative sample size. Considering the importance of diabetes as a main cause of disability and with the view of the fact that this disease can be effectively controlled, we aimed to conduct a study to shed some light on the prevalence of diabetes and its related risk factors in professional drivers. Moreover, we aimed to investigate the known risk factors of this disease in order to better clarify the burden of this health issue in Iran.

## Methods

The current study is a cross-sectional descriptive -analytic study. The samples included 1903 professional drivers who referred to one of the authorized occupational health clinics in Tehran between 2010 and 2011. All of the drivers who received health card enrolled in our study, so the drivers who had DM type 1(insulin dependent, genetic type) exclude. Prior to conducting the study, informed consent was obtained from all drivers. Respondent’s anonymity was assumed. All blood samples were collected in the morning following 8 hours of fasting.

Basic demographic information as well as the height, weight, the class of the driving license (car or heavy vehicle), blood pressure, blood sugar levels, triglyceride and cholesterol levels were recorded. Then, the forms were completed by the medical practitioner who was in charge of data collection.

The collected information then was analyzed using different descriptive and analytic methods with the use of the SPSS software version 14. For comparison of quantitative variables *T*-test, and for qualitative parameters Kai-2 test was utilized. In all the analyses, P value less than 5% was considered significant. The definitions of different medical conditions and diseases were extracted from the Harrison Textbook of Medicine 18th edition.

## Results

In the current study, 1903 drivers comprised of both car and heavy vehicle drivers were assessed in terms of diabetes and its risk factors. Amongst the participants, 52.8% had class 1 driving licenses (for cars) and 47.2% had class 2 licenses (for heavy vehicles). The youngest person was 21 years old and the oldest was 69. The mean age was 41.55 ± 10.53 years. Participants were divided into age groups of 10 years period and the majority belonged to the group 40–49 years (28.8%).

47.8% of the participants had normal blood sugar levels (FBS < 100 mg/dl). Hyperglycemia (FBS ≥ 100) was detected in 52.1% of them amongst whom 43% were in the pre-diabetic stage (100 < FBS < 126 mg/dl) and 9.1% were diabetic (FBS ≥ 126 mg/dl).

The mean age of the diabetic participants was 48.9 years and that of the others was 40.83 years. The prevalence of diabetes in those who were older than 40 was higher in comparison with the younger participants (odds ratio: 4.25; 95% CI:3.01 to 6.78; P < 0.001)

Excess weight (BMI > 25 kg/m^2^) was seen in 1226 subjects (65/6%) amongst whom 837 (44/8%) were overweight (25 < BMI < 29.9) and 389 (20.8%) were obese (BMI ≥ 30). It is noteworthy that excess weight was increased with age (P < 0.001).

Hyperglycemia in the overweight subjects was 1/96 times more common in comparison with the normal individuals (odd ratio: 1.96; 95% CI: 1.34 to 2.84).

The prevalence of hypertension (BP ≥ 140/90 mmHg) was 16.4% which was increased with age (P < 0.001). The mean age of those with hypertension was 47/7 and that in those with normal blood pressure was 40/4 years (P < 0.001).

Excess weight (BMI ≥ 25 kg/m^2^) was another risk factor for increased blood pressure. 65/6% of individuals with high blood pressure simultaneously had excess weight as well and 34/4% had normal BMI (odd ratio: 2.12; 95% CI: 1.58 to 2.84; P < 0.001).

The mean of BMI in those without hypertension was 26.5 kg/m^2^ and that in the subjects with hypertension was 28.4 kg/m^2^ (P < 0.001).

High blood pressure in hyperglycemic individuals was 35% in comparison with 14.4% which was recorded in those with normal glucose levels.

Table 1 showes the prevalence of hyperglycemia in the study population with regard to risk factors. In this table risk factors included high blood pressure (BP ≥ 140/90 mmHg), excess weight (BMI ≥ 25 Kg/m^2^), hypertriglyceridemia (Triglycerids ≥ 150 mg/dl), and hypercholesterolemias (Total Cholesterol ≥200 mg/dl) which are compared in two hyperglycemic (FBS ≥ 100 mg/dl) and normoglycemic (FBS < 100 mg/dl) groups.

**Table 1:** The prevalence of hyperglycemia in the studied subjects with regard to the risk factor

**Diabetes risk factor**	**Hyperglycemic**	**Normoglycemic**
Hypertension	203 (67%)	100 (33%)
Excess weight	705 (57.8%)	515 (42.2%)
Hypercholesterolemia	477 (60.3%)	314 (39.7%)
Hypertriglyceridemia	534 (58.5%)	379 (41.5%)
No risk factor	140 (46.8%)	159 (53.2%)

Table 2 compares BMI, age, systolic blood pressure, diastolic blood pressure, and triglyceride and cholesterol levels of the study subjects in three groups, namely normal, pre-diabetic, and diabetic individuals. In this table, P-value of each of the mentioned parameters is calculated once by comparison of normoglycemic group with the other two groups and once more by comparison of the pre-diabetics with diabetic group. The differences in all parameters were found to be significant albeit to different extents.

**Table 2:** The prevalence of risk factors in the three groups

	**Diabetes**	**Pre diabetes**	**Normal**
Number (%)	173(9.1%)	815(43%)	906(47.8%)
FBS	166.21 ± 52.30	108.98 ± 6.25	92.46 ± 6.09
BMI(kg/m^2^)	27.97 ± 4.23 ^b2^	27.01 ± 4.22	26.50 ± 4.07
BPs(mmHg)	128.30 ± 16.27 ^c3^	121.55 ± 13.58	118.77 ± 13.17
BPd(mmHg)	81.26 ± 8.13 ^b3^	79.47 ± 8	78.07 ± 6.98
Triglyceride	240.39(44–1100) ^c3^	177.08(32–966)	163.20(35–801)
Total cholesterol	199.26 ± 42.87 ^b3^	196.88 ± 44.28	187.89 ± 40.98
Age(yr)	48.92 ± 8.58 ^c3^	41.93 ± 10.42	39.61 ± 10.5

In diabetic individuals, HbA1c was also measured. This index is considered as another criterion for diagnosis of diabetes. The levels of HbA1c in those with blood sugar higher than 126 were as follows: 77/6% of the subjects with this criteria were categorized in the diabetic category (HbA1C ≥ 6.5%), 13.8% were in the pre-diabetic state (5.6 < HbA1c < 6.5) and 8.6% of them were in the normal group (HbA1c ≤ 5.6%).

## Discussion

9.1% of our subjects had blood sugar ≥ 126. Prevalence of diabetes in professional drivers in Hong Kong is reported to be 8.1% [10]. In one study carried out on truck and bus drivers in Kashan, this prevalence was demonstrated to be 7% [11]. Therefore, it can be asserted that these findings seem to be in accordance with each other. However, it may be postulated that this prevalence can vary in different populations considering diet and genetics in various regions of the world [1].

44.8% of the study individuals were overweight (25 < BMI < 29.9) and 20.8% were obese (BMI ≥ 30). Cumulatively, 65.6% of our study population had excess weight. In one study which was performed in Kashan on bus and truck drivers, it was observed that 41% and 23.1% of the drivers were overweight and obese respectively (64.1% combined) [11]. In one study carried out in Poland, this rate was 62% [14] and in another study in Mexico, it was reported to be 75.2% [15]. In another study conducted by Metabolism and Endocrine Glands Institution at medical university of Shahid Beheshti in Iran, the prevalence of overweight and obesity in the Tehran population was reported to be 35% and 23.7% [16]. A simple comparison demonstrates that our findings are in congruence with the findings of other studies. However, an interesting finding was that our results showed that the average weight of drivers was more than the general population of Tehran. In general population, the incidence of excessive body weight increase with age [17-19], this finding was also seen in our study.

Our findings indicated an increased prevalence of carbohydrate metabolism disorders in those with excess weight. The risk of hyperglycemia in this cohort was approximately two-fold (1.96) in comparison with the normal individuals. In a different study, this risk was found to be slightly more than two-fold (2.03) [14].

High blood pressure is considered as another risk factor for diabetes and its prevalence was found to be 16.4% in our study population. However, it should be mentioned that the diagnosis of hypertension cannot be made on the basis of only one single measurement [1,20]. Considering this, in our study the term “increased blood pressure” is used instead of hypertension. In one study in 400 different cities, it was demonstrated that the prevalence of a blood pressure higher than 140/90 mmHg in initial screening was 65.8% while the diagnosis of hypertension was made later in 76.1% of them [21].

In our study, the prevalence of increased blood pressure was increased with increasing age, a finding which has been reported in other studies [14].

Hypertension in combination with excess weight was seen in more than two-fold (OR = 2.12) of individuals in comparison with those who had high blood pressure with normal weight. According to the findings of Polish study, this was reported 4-folds [14].

These findings showed that obesity could be a risk factor for hypertension [22]. The prevalence of excess weight and obesity in those with high blood pressure in our study was 65.6%. This prevalence has been reported 68% in Poland [23], 72.6% in Sweden [24], and 77% in Greece [25]. It can be concluded that our findings were in congruence with the findings of other studies in different countries.

In our study, the prevalence of hypertension was 35% in hyperglycemic individuals in comparison with 14.4% in the normoglycemic population. In one study, these figures were 61.7% and 38.3% respectively [14], which is the same as other study that reported these results as 68.6% and 48% respectively [26]. A different study demonstrated slightly higher prevalence (85.3 versus57.9%) [27] which can also be considered to be in accordance with our findings. However, the present study noted lower prevalence of high blood pressure in hyperglycemic individuals, this may be due to higher average age of participants in other studies, since the age itself is an independent risk factor for both hypertension and hyperglycemia.

High triglyceride levels were detected in 28.3% of the drivers in this study and high cholesterol levels were reported in 41.8%. A study in Iran noted high triglyceride and cholesterol levels as 53.4% and 35.4% respectively [11], also similar study in Taiwan, high triglyceride and cholesterol levels were seen in 69.4% and 34.4% of the participating drivers [28].

The reason for the observed difference in triglyceride and cholesterol levels can be claimed to be the difference in the age, sex, nutritional habits, and different diagnostic criteria.

In our study, age, BMI, blood pressure, and triglyceride and cholesterol levels were significantly higher in hyperglycemic individuals in comparison with the normoglycemic subjects. The prevalence of diabetes increases with age, and moreover, most people with type 2 diabetes, even with good glycemic control, have dyslipidemia. As dyslipidemia, hypertension, and obesity are all predisposing factors for diabetes [1]. The findings of present study were the same.

In our study, 77.6% of those who were considered as diabetic based on the blood sugar level were also categorized as diabetic based on the measurement of HbA1c. 13.8% of these individuals were in pre-diabetes stage and 8.6% were considered normal. This diagnosis based on two different criteria can be considered as completely accurate and valid and is used for the diagnosis of diabetes in different studies [1].

One of the limitations of the current study can be claimed to be its cross-sectional design. It is demonstrated that it is difficult to establish a cause-effect relationship in studies with such designs. Another limitation of the study can be claimed to be due to the fact that the measurement of blood pressure and blood sugar levels were only performed once and it is not possible to make the diagnosis of hypertension or diabetes merely on the basis of one single measurement of blood pressure and blood sugar levels respectively. Therefore, in this study, the terms “high blood pressure and high blood sugar” are used instead of hypertension or diabetes. It is noteworthy, that our data was gathered during periodic medical examination of drivers to renew their driving license, and it was not possible to collect more informations.

The large study cohort can be considered as strength of this study. Measurement of different risk factors for diabetes and assessment of their relationship with one another, which is not usually performed, can also be considered as strength of the study.

In conclusion, this study highlights the increasing prevalence of type 2 diabetes and its risk factors in Iranian drivers in comparison with the normal population, it is of utmost importance to carry out more screening investigations in this regard. Moreover, with regard to the potential influence of the performance of a driver on public safety as a whole as well as the economy of the country, it is of utmost importance to investigate, intervene, and modify lifestyle of drivers. A good approach for professions associated with public safety, systematic referral to to occupational medicine services and providing prophylactic activities.

## Competing interest

The authors declare that they have no competing interests.

## Authors’ contributions

NI: design, analysis of study,and final approval of manuscript. MM: conception, drafting the manuscript, final approval of manuscript. OA: interpretation of data, final approval of manuscript. MS: design, acqusition of data, final approval of manuscript. All authors read and approved the final manuscript.
